# AhR Activation by TCDD (2,3,7,8-Tetrachlorodibenzo-p-dioxin) Attenuates Pertussis Toxin-Induced Inflammatory Responses by Differential Regulation of Tregs and Th17 Cells Through Specific Targeting by microRNA

**DOI:** 10.3389/fmicb.2019.02349

**Published:** 2019-10-18

**Authors:** Zinah Zamil Al-Ghezi, Narendra Singh, Pegah Mehrpouya-Bahrami, Philip Brandon Busbee, Mitzi Nagarkatti, Prakash S. Nagarkatti

**Affiliations:** Department of Pathology, Microbiology and Immunology, University of South Carolina School of Medicine, Columbia, SC, United States

**Keywords:** aryl hydrocarbon receptor (AhR), pertussis toxin, 2, 3, 7, 8-Tetrachlorodibenzo-p-dioxin, immunosuppression, inflammation, microRNA

## Abstract

The Aryl Hydrocarbon Receptor (AhR) is a transcription factor that, when activated by ligand-binding, has been shown to regulate the immune response. Pertussis Toxin (PTX) is a virulence factor found in *Bordetella pertussis*, a human respiratory pathogen that causes whooping cough. PTX promotes colonization and disease promotion by triggering a heightened inflammatory response. The role of AhR in the regulation of PTX-mediated inflammation has not previously been studied. In the current study, we investigate if AhR activation by 2,3,7,8-Tetrachlorodibenzo-p-dioxin (TCDD), a well characterized ligand, can attenuate PTX-mediated systemic inflammation. To that end, C57BL/6 mice were injected intraperitoneally (IP) with PTX twice and treated with TCDD or vehicle (VEH). The PTX+VEH group showed elevated levels of pro-inflammatory cytokines (IL-17A, IL-6, and IFNγ) in serum and increased proportions of CD4+ Th1 and Th17 cells in their spleens. In contrast, the PTX+TCDD group showed significantly lower levels of these inflammatory cytokines and decreased proportions of Th1 and Th17 cells, but increased proportions of Th2 and FoxP3+Tregs when compared to the PTX+VEH group. PTX+TCDD treated mice also showed elevated levels of IL-10, and TFG-b, potent anti-inflammatory cytokines. MicroRNAs (miRs) analysis of CD4+ T cells from the spleens of the PTX+TCDD treated mice revealed significant alterations in their expression and several of these miRs targeted cytokines and signaling molecules involved in inflammation. Specifically, the PTX+TCDD group had a significantly enhanced expression of miR-3082-5p that targeted IL-17, and a decreased expression of miR-1224-5p, which targeted FoxP3. Transfection studies with these miR mimics and inhibitors confirmed the specificity of the target genes. The current study suggests that AhR activation by TCDD suppresses PTX-induced inflammation through miR regulation that triggers reciprocal polarization of Tregs and Th17 cells and also suggests that AhR activation may serve as a treatment modality to suppress heightened inflammation induced during *B. pertussis* infection.

## Introduction

TCDD (2,3,7,8-Tetrachlorodibenzo-p-dioxin), also known as dioxin, is a polyhalogenated aromatic hydrocarbon (Birnbaum, 1994). TCDD is a potent agonist with high binding affinity to the aryl hydrocarbon receptor (AhR), which is extensively used to study the impact of AhR activation on various physiological and immune functions (Baccarelli et al., 2004; Singh et al., 2007). AhR belongs to the basic helix-loop-helix–PER-ARNT-SIM transcription factor family and is a cytosolic-localized receptor. Studies have shown that in addition to its toxicological functions, AhR is known to regulate the immune response, particularly through affecting T cell differentiation (Quintana and Sherr, 2013; Gutierrez-Vazquez and Quintana, 2018). Upon binding to known ligands, such as TCDD, AhR translocates to the nucleus and dimerizes with the aryl hydrocarbon receptor nuclear translocator (ARNT), and the AhR-ARNT complex initiates the transcription of genes with promoters containing a dioxin-responsive element (DRE) consensus sequence (DeGroot and Denison, 2014; Li et al., 2014). In addition to the well characterized ligand TCDD, a variety of other compounds including tryptophan derivatives, dietary flavonoids, and biphenyls have been shown to bind to AhR with varying affinities (Busbee et al., 2013). While AhR was initially discovered in the context of activation by environmental chemicals such as TCDD, leading to the induction of xenobiotic metabolizing enzymes, and regulating the toxicity mediated by such chemicals, more recent studies have shown that AhR activation plays diverse roles in cellular functions, especially in the regulation of T cell differentiation (Rothhammer and Quintana, 2019).

Pertussis Toxin (PTX) is one of the most important virulence associated factors of *Bordetella pertussis*, the gram-negative bacterium that is responsible for whooping cough. PTX is a bacterial toxin belonging to the A-B structure class, and when the B subunit binds to receptors on the cell surface, subunit A interrupts intracellular signaling *via* irreversible ADP-ribosylation of the Gi subclass of G protein (Burnette, 1997; Falnes and Sandvig, 2000). PTX exerts its effects through binding to G protein coupled receptors, and because they are present in several mammalian cell types, it affects most cells types (Lahiani et al., 2017). One of the G_i/o_ protein dependent effects of PTX is lymphocytosis (Sato et al., 1974; Morse and Morse, 1976). Moreover, PTX also acts *via* a phosphokinase C pathway to increase the permeability of the blood-brain barrier resulting in neurological effects (Bruckener et al., 2003). Studies have shown that PTX can trigger the development of Th17 cells that promote inflammation (Chen et al., 2007; Hofstetter et al., 2007; Andreasen et al., 2009). PTX is also recognized as a major contributor to autoimmune pathogenesis (Chen et al., 2007). Previous studies have reported increased interferon gamma (IFN-γ) secretion by immune cells in response to PTX (Vermeulen et al., 2010). In addition, the upregulation of interleukin-17 (IL-17) by PTX during the peak of *B. pertussis* infection leads to the increased infiltration of neutrophils in lung airways. Several studies comparing wild-type and PTX-deficient *B. pertussis* strains have revealed that PTX plays an important role in the promotion of an infection in the respiratory tract, through an initial phase of immune suppression followed by enhanced inflammation, finally, leading to lung pathogenesis (Khelef et al., 1994; Carbonetti, 2015, 2016). Thus, agents that suppress inflammation induced by PTX may serve as treatment modalities.

MicroRNAs (miRs) are short non-coding single stranded RNAs, about 19–25 nucleotides long, that negatively regulate target genes’ expression at the post transcriptional level (Christensen and Schratt, 2009; Hou et al., 2011). A connection between microRNAs and different diseases, such as inflammatory bowel disease, autoimmune diseases, and cancers, are being investigated (Christensen and Schratt, 2009; Sonkoly and Pivarcsi, 2009; Raisch et al., 2013). Recent studies have shown that exposure to chemicals can cause alterations in miRNAs and gene expressions that lead to different health problems and diseases (Fukushima et al., 2007; Hou et al., 2011). The evidence linking environmental chemical contaminants like dioxin and miRNAs functions to human diseases is rapidly growing (Hou et al., 2012). However, it is not yet clear how AhR activation by TCDD alters miRNAs or the possibility that TCDD-induced miRNAs may control mRNA that regulate inflammation. Some studies have confirmed an association between deregulation of miRNAs and exposure to environmental chemicals, and dioxins are among them (Guida et al., 2013). It has been found that the toxic effects of TCDD may also be controlled by certain epigenetic mechanisms like DNA methylation or histone modification (Patrizi and Siciliani de Cumis, 2018). The involvement of PTX in miRNAs dysregulation is also not fully understood and studies in this field are still limited. In one study, it was shown that miR-202, 342-5p, 206, 487b, 576-5p were upregulated in pertussis patients (Ge et al., 2013).

The role of AhR activation on inflammation induced by PTX has not been previously studied. In this study, we investigated whether AhR activation by TCDD can attenuate PTX-induced inflammation in mice and if so, whether such anti-inflammatory action is mediated by miRNAs. Our studies demonstrate that TCDD does alter the expression of several miRNAs that target various cytokine and transcription factors in T cells, leading to the suppression of PTX-mediated inflammation.

## Materials and Methods

### Mice

Female C57BL/6 mice (6–8 weeks old) were purchased from Jackson Laboratories (Indianapolis, Indiana). The animals were housed in the AALAC approved animal facility at the School of Medicine, of the University of South Carolina.

### Ethics Statement

Animals used in the experiments of this study were approved by the Institutional Animal Use and Care committee of the University of South Carolina.

### PTX and TCDD Administration

TCDD was kindly provided by Dr. Steve Safe (Institute of Biosciences & Technology, Texas A&M Health Science Center, College Station, TX, United States). TCDD was dissolved in 100% DMSO (Sigma, St. Louis, MO, United States) after which, 10 μg/ml of the TCDD stock was further diluted with corn oil (CO) (Sigma, St. Louis, MO, United States) (final concentration: 100 μg/ml). The final concentration of DMSO in the corn oil was 2% (Singh et al., 2012). Groups of 3–5 mice were first injected IP with vehicle (CO), or TCDD (25 μg/kg body weight) then injected IP with 400 ng/mouse PTX (List Biological Laboratories, Campbell, CA, United States). Twenty-four hour later, the mice were rechallenged with the same dose of PTX intraperitoneally, as described (Elliott et al., 2018).

### Determination of Cytokine Expression by Performing ELISA

To examine the expression of pro- and anti-inflammatory cytokines post-PTX and Vehicle (corn oil) or TCDD treatments, blood was collected from a control corn oil vehicle (VEH), PTX+VEH and PTX+TCDD mice. Sera was extracted and either used immediately or stored at −20°C until further use. Sera was exposed to one thaw cycle and cytokines of interest, such as IL-17A, IL-6, IFNγ, and IL-10 were determined by performing ELISA using BioLegend ELISA Max kits (BioLegend, San Diego, CA, United States) following the protocol of the company and as described in details previously by Busbee et al. (2014).

### Flow Cytometry Analysis of Splenic Cells Post-PTX and TCDD Treatment

Flow cytometry was used to determine various cell types present in the spleens of mice treated with PTX+ Vehicle and PTX+TCDD. In brief, single cell suspensions of spleens from both groups were first prepared. The splenic cells were then treated with GolgiPlug purchased from BD (BD Biosciences, San Jose, CA, United States) for at least 6 h at 37°C, to enhance cytokine staining. Cells treated with Fc block for 10 min at 4°C, were then stained with surface antibodies using PE-conjugated anti-mouse CD4 (clone GK1.5) for 30 min at 4°C. Next, the cells were washed with cold PBS twice and prepared for intranuclear staining using the True-Nuclear Transcription Factor Buffer kit (BioLegend, San Diego, CA, United States) or intracellular staining with a Fixation/Permeabilization Solution Kit from BD Biosciences (San Jose, CA, United States) as per the instructions of the manufacturers. The following Abs were used: anti-FoxP3, anti-IL-10, anti-TGF-β, anti-IL-17, and anti-IFN-γ (BioLegend, San Diego, CA, United States). For MDSCs, splenocytes were stained with fluorescent conjugated antibodies: CD11b and Gr-1. For MDSC subsets, additional surface staining with anti-Ly6G or anti-Ly6C were used to differentiate between granulocytic and monocytic subsets, respectively. Stained cells were analyzed using BD FACSCelesta (BD Biosciences, San Jose, CA, United States) and DIVA software or FlowJo software.

### Investigation of the miRNA Profile in Splenic CD4+ T Cells

To investigate the miRNA profile in CD4+ splenic cells, CD4+ cells were isolated from the splenocytes of the PTX+VEH and PTX+TCDD groups of mice using the EasySep PE Positive Selection Kit (STEMCELL Technologies, Cambridge, MA, United States) and anti-CD4 mAb (BioLegend, San Diego, CA, United States). The isolated CD4 cells were washed with cold PBS and then QIAzol Lysis Reagent (Qiagen, Germantown, MD, United States) was added and either used immediately for total RNA extraction including miRNA or stored at −80°C for future use. Total RNA including miRNAs were extracted using a miRNeasy micro kit (Qiagen, Germantown, MD, United States) according to the manufacturer’s instructions. The concentration and purity of the extracted RNAs was determined using the NanoDrop 2000 spectrophotometer (Thermo Scientific, Wilmington, DE, United States). The expression profile of miRNAs was determined by performing microRNA arrays using the Affymetrix miRNA array version 4 (JHMI Deep sequencing & Microarray Core) and GeneChip miRNA 3.0-array platform. miRNA data were analyzed using Ingenuity Pathway Analysis (IPA, Qiagen) as described (Alharris et al., 2018). In this study, a >1.5-fold change in miRNA expression was considered positive (Elliott et al., 2016).

### Quantitative Real-Time PCR (QRT-PCR) to Determine the Expression of miRNA and Genes of Interest

Next, we validated several miRNAs that were selected based on their expression and corresponding gene expressions. For miRNA expression analysis, cDNA synthesis was performed from total RNA using the miScript II cDNA Synthesis Kit (# 218161, Qiagen, Germantown, MD, United States). Two step miRNA qRT-PCR was carried out using SsoAdvanced SYBR green Mix (#1725270 Bio-Rad, Hercules, CA, Germantown) with mouse primers for SNORD96A (#MS00033733 Qiagen, Germantown, MD, Germantown), miR-141-3p (#MS00001610 Qiagen, Germantown, MD), miR-142-3p (# MS00012201 Qiagen, Germantown, MD, Germantown), and miR-211-3p (# MS00024563 Qiagen, Germantown, MD, Germantown). Expression levels for miRNA were normalized to SNORD96A. Specific primers sequences are provided in Table 1. Furthermore, we performed qRT-PCR to determine the expression of miR-1224-5p (Qiagen # MS00011074) and miR-3082-5p (Qiagen # MS00025102) that target FoxP3 and IL17 genes, respectively. The selection of these two miRNAs was based on their alignment with highly conserved regions of their target genes using miRNA.org, miRWalk, and TargetScan software. We also evaluated expression of target genes (IL-10, TGF-β1, TGFβ2, TGFβR3, TGFβR1, GATA3, SMAD2, IL-6, and, RORγt), as described (Alghetaa et al., 2018). For gene expression analysis, cDNAs was generated as described above from total RNA using the miScript II cDNA synthesis kit. PCR was performed with SsoAdvanced SYBR green mix with mouse primers for IL-10, TGF-β1, TGFβ2, TGFβR3, TGFβR1, GATA3, SMAD2, IL-6, and RORγt customized and ordered from IDT (Coralville, IA, Germantown). All PCR experiments used a CFX96 Touch Real-Time PCR Detection System (Bio-Rad, Hercules, CA, Germantown), and expression levels were normalized to GAPDH mRNA levels. Fold changes were calculated using the 2^–ΔΔCT^ method. The specific primer sequences of the genes are provided in Table 2.

**TABLE 1:** Up-regulated and down-regulated miRNAs upon TCDD exposure.

**microRNA Identification**	**microRNA Sequence (5′-3′)**
mmu-miR-211-3p	GCAAGGACAGCAAAGGGGGGC
mmu-miR-142-3p	UGUAGUGUUUCCUACUUUAUGGA
mmu-miR-141-3p	UAACACUGUCUGGUAAAGAUGG
mmu-miR-3082-5p	GACAGAGUGUGUGUGUCUGUGU
mmu-miR-1224-5p	GUGAGGACUGGGGAGGUGGAG

**TABLE 2:** mRNA related oligonucleotides that used in this study.

**Gene**	**Forward Sequence (5′–3′)**	**Reverse Sequence (5′–3′)**
Tgfβ1	CTCCCGTGGCTTCTAGTGC	GCCTTAGTTTGGACAGGATCTG
Tgfβ2	CTTCGACGTGACAGACGCT	GCAGGGGCAGTGTAAACTTATT
Tgfβr1	TCTGCATTGCACTTATGCTGA	AAAGGGCGATCTAGTGATGGA
Tgfβr3	GGTGTGAACTGTCACCGATCA	GTTTAGGATGTGAACCTCCCTTG
IL10	CCCATTCCTCGTCACGATCTC	TCAGACTGGTTTGGGATAGGTTT
Foxp3	CCCATCCCCAGGAGTCTTG	ACCATGACTAGGGGCACTGTA
Smad2	ATGTCGTCCATCTTGCCATTC	AACCGTCCTGTTTTCTTTAGCTT
GATA3	CTCGGCCATTCGTACATGGAA	GGATACCTCTGCACCGTAGC
IL17a	TTTAACTCCCTTGGCGCAAAA	CTTTCCCTCCGCATTGACAC
RORγt	GACCCACACCTCACAAATTGA	AGTAGGCCACATTACACTGCT
IL6	CCAAGAGGTGAGTGCTTCCC	CTGTTGTTCAGACTCTCTCCCT
GAPDH	TGGATTTGGACGCATTGGTC	TTTGCACTGGTACGTGTTGAT

### Transfection of T Cells With miR-1224-5p and miR-3082-5p Mimic or Inhibitor

Two miRNAs (miR-3082-5p and miR-1224-5p) were selected based on their alignments with mouse IL-17 and FoxP3 genes. For transfection, we used T cells purified from naïve splenocytes and maintained for 24 h in complete RPMI 1640 medium, supplemented with 10% heat-inactivated fetal bovine serum, 10 mm L-glutamine, 10 mM HEPES, 50 μm β-mercaptoethanol, and 100 μg/ml penicillin/streptomycin at 37°C and 5% CO_2_ (Singh et al., 2012). Prior to transfection, T cells were seeded at a density of 2 × 105 cells/well in a 24-well plate and activated with 1 μg/ml of Staphylococcus enterotoxin B (SEB). The following day, the cells were transfected with scrambled miRNA (Mock) control or mature miR-1224-5p mimic (Qiagen # MSY0005460), miR-1224-5p inhibitor (Qiagen # MSY0005460), mature miR-3082-5p mimic (Qiagen # YM00470236), or miR-3082-5p inhibitor (Qiagen # YI04103580) using the HiPerFect Transfection Reagent (Qiagen, Germantown, MD, United States) and following the protocol of the company.

### Statistical Analysis

We used GraphPad Prism 6 software to perform statistical analyses. In direct comparisons between two groups, two-tailed Student’s-*t* tests were performed. In comparisons with more than two groups, One-way ANOVA with a Bonferroni *post hoc* correction was performed. All experiments were repeated at least twice, unless otherwise indicated in each figure legend, and a *p* value < 0.05 was considered significant.

## Results

### TCDD Suppresses PTX-Induced Inflammation in Mice

**FIGURE 1:**
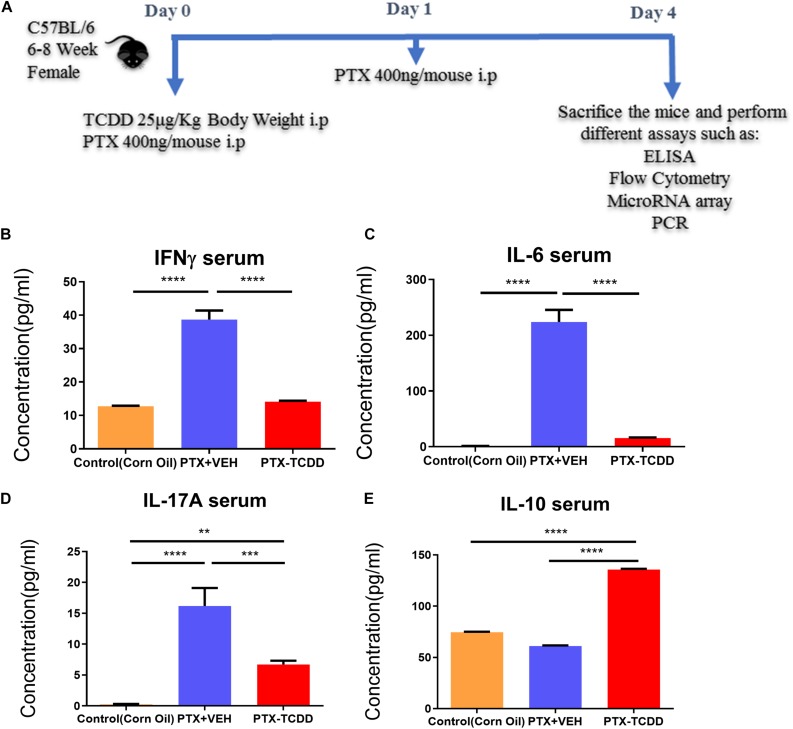
TCDD suppresses PTX-induced inflammation in mice. Control mice (corn oil) or mice injected with PTX and TCDD or VEH as shown in panel **(A)** and 4 days later; Control, PTX+VEH or PTX+TCDD groups were investigated for inflammation. **(B)** ELISA was performed using sera collected from the PTX+VEH and PTX+TCDD group of mice. Cytokines IFNγ **(B)**, IL-6 **(C)**, IL-17A **(D)**, and IL-10 **(E)** were measured. Data are expressed as the mean ± SEM and statistical significance analyzed using Student’s *t*-test. Is indicated as ^∗∗^*p* < 0.01, ^∗∗∗^*p* < 0.001, and ^****^P < 0.0001 between the two groups.

In this study, we investigated the effect of AhR activation by TCDD on the PTX-mediated inflammatory response in C57BL/6 mice. To that end, mice received a VEH consisting of corn oil, PTX+TCDD or PTX+Veh on day 0, and 24 h later (day 1), all mice received a second dose of PTX. On day 4, the mice were euthanized and studied for inflammation (Figure 1A). First, we performed ELISA using sera from the control (corn oil only), PTX+VEH, and PTX+TCDD groups. The data showed that there were significant increases in pro-inflammatory cytokines IL-17A, IFN-γ, and IL-6 in the PTX+VEH group when compared to the vehicle controls, however, the PTX+TCDD group showed a significant decrease in these inflammatory cytokines when compared to the PTX+VEH group (Figures 1B–D). In addition, there was a significant increase in IL-10, an anti-inflammatory cytokine, in the PTX+TCDD groups when compared to either the control or the PTX+VEH groups (Figure 1E). These data demonstrated that PTX induces a potent inflammatory cytokine response and TCDD suppresses this response while enhancing anti-inflammatory cytokine, IL-10.

### TCDD Promotes Differential Regulation of Tregs and Th17 Cells and Generation of MDSCs *in vivo*

**FIGURE 2:**
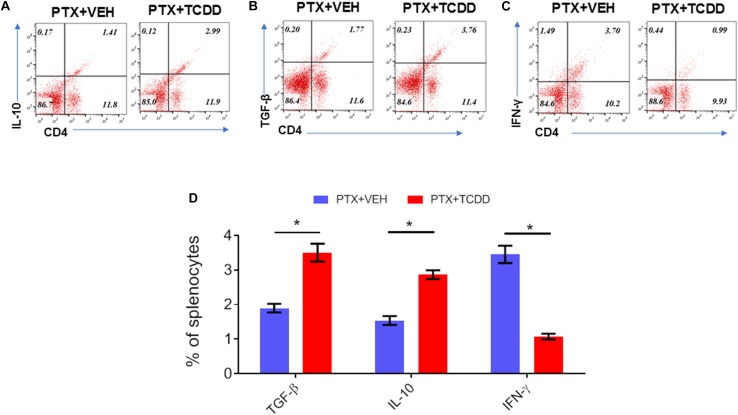
TCDD treatment suppresses PTX-induced Th1 cells and promotes Th2 cells. Mice were immunized with PTX and injected with TCDD as described in the legend of Figure 1. Splenocytes were double-stained for CD4 and various cytokines and analyzed by flow cytometry: **(A)** IL-10; **(B)**TGF-β; **(C)** IFN-γ. Panels **A–C** show a representative experiment and data from multiple experiments plotted in panel **D**. Statistical analysis was performed using Student’s *t*-test. Data are expressed as the mean ± SEM and statistical significance between the two groups is indicated as ^∗^*p* < 0.05.

**FIGURE 3:**
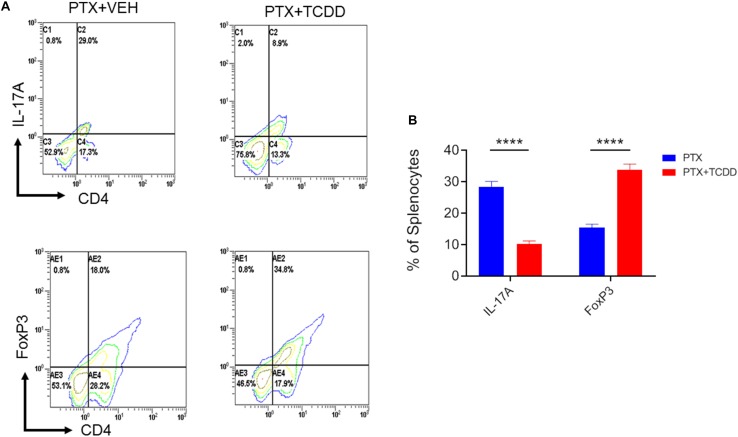
TCDD promotes Treg while inhibiting Th17 induction. Mice were immunized with PTX and injected with TCDD as described in the legend of Figure 1. Splenocytes were double-stained for CD4 and FoxP3 or CD4 and IL-17 markers and analyzed using flow cytometry. Panel **A** shows a representative experiment while data from multiple experiments is plotted in panel **B**, showing reciprocal regulation of Th17 vs. Tregs between the two groups. Statistical analysis was performed using Student’s *t*-test with the differences between the two groups showing ^****^p < 0.0001.

Next, we investigated the phenotypic changes in splenocytes harvested from PTX+VEH and PTX+TCDD mice. We analyzed for the following cell types: Tregs: CD4+FoxP3+, Th17: CD4+IL17+, Th1: CD4+IFNγ+, and Tr1:CD4+IL10+, and CD4+TGFβ+ cells. We noted that the percentage of Tr1 cells (CD4+IL10+ cells and CD4+TGFβ+) were significantly increased in the PTX+TCDD group, when compared to the PTX+VEH group (Figures 2A,B,D). In contrast, the proportions of Th1 cells was significantly decreased in the PTX+TCDD group when compared to controls (Figures 2C,D). Th17 and Tregs are reciprocally regulated and thus when we analyzed these cells in the two groups we found that the percentage of Th17 cells (CD4+IL17+) were increased while that of Tregs (CD4+FoxP3+) were decreased in the PTX+VEH group and, interestingly, this pattern was completely reversed in the PTX+TCDD group (Figures 3A,B).

**FIGURE 4:**
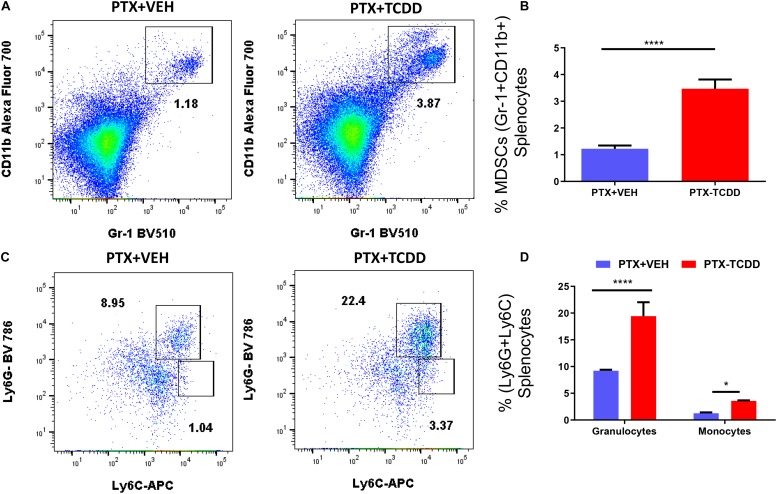
TCDD induces MDSCs: Mice were immunized with PTX and injected with TCDD as described in the legend of Figure 1. Spleen cells were double-stained for Gr-1+CD11b+ cells to determine MDSC percentages. Panel **A** shows a representative experiment while data from multiple experiments are plotted in panel **B**. Percentages of MDSC subsets (granulocytic, CD11b+Ly6G+, and monocytic, CD11b+Ly6C+) were also determined, with representative data presented in panel **C** and data from multiple experiments represented in panel **D**. Statistical analysis was performed using a Student’s *t*-test. In panels **C,D** data are expressed as the mean ± SEM and statistical significance is indicated as ^∗^*p* < 0.05 or ^****^p < 0.0001 when the two groups are compared.

We also examined myeloid-derived suppressor cells (MDSCs; CD11b+/Gr1+) and MDSC subsets (monocytic CD11b+Ly6C+ and granulocytic CD11b+Ly6G+) in the spleens of the PTX+VEH and PTX+TCDD mouse groups because these cells are also highly immunosuppressive and have been known to induce Tregs (Hegde et al., 2013; Jitschin et al., 2014). There were significant increases in the percentage of MDSCs in mice exposed to PTX+TCDD compared to PTX+VEH (Figures 4A,B). In addition, both granulocytic and monocytic MDSC percentages increased after TCDD injection in PTX-exposed mice (Figures 4C,D), although there was more induction of granulocytic MDSCs when compared to monocytic MDSCs.

### TCDD Dysregulates Expression of miRNAs in Splenic Cells *in vivo*

**FIGURE 5:**
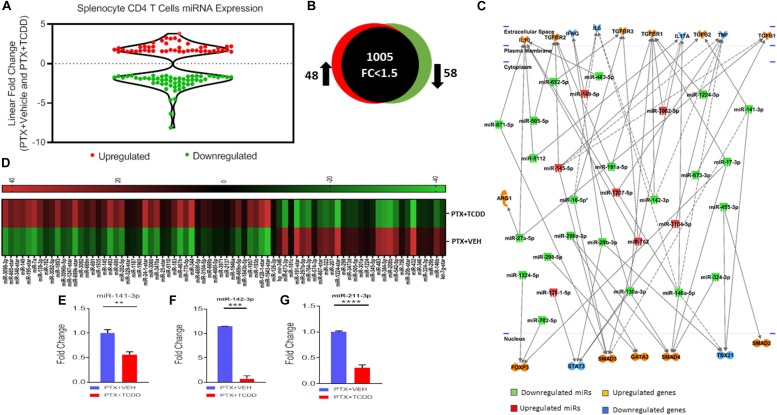
MicroRNAs Analysis: Mice were immunized with PTX and injected with TCDD as described in the legend of Figure 1. Differentially expressed miRNAs were analyzed in splenic CD4+ T cells. Total RNA was isolated from CD4+ T cells obtained from the spleen of the PTX+VEH or PTX+TCDD groups (*n* = 5). A miRNA microarray assay was performed to test differentially expressed miRNAs. **(A)** The fold change distribution of the miRNAs found within CD4+ T cells from the spleens of the PTX+Vehicle and PTX +TCDD mice. **(B)** Proportional Venn diagram illustrating fold change (>1.5) of miRNAs between the two groups. **(C)** Inflammatory pathway analysis of miR mediating dysregulation in gene expression in PTX+TCDD group. **(D)** Heat map of hierarchical clustering of the relative expression of miRNA alterations. The color scale denotes those miRNAs that were upregulated (red) and downregulated (green). **(E–G)** Expression levels of select downregulated miRNAs analyzed by qRT-PCR using Snord96a as a control.

To understand the anti-inflammatory/immunosuppressive effects of TCDD, we investigated the molecular mechanisms that TCDD may regulate to affect gene expression. To this end, we performed miRNA arrays using total RNA from splenic CD4+ T cells from the PTX+VEH and PTX+TCDD groups as described in the section “Materials and Methods.” Of approximately 1,111 miRNAs tested, 157 miRNAs were differentially expressed (Fold change >±1.5) (Figure 5A). A proportional Venn diagram was generated to represent the fold change of 1,111 miRNAs in the CD4+ T cells examined, showing that there were 48 miRNAs that were upregulated (>1.5-fold) and 58 miRNAs that were downregulated (>1.5-fold) in splenic CD4 T cells of the PTX+TCDD group of mice, when compared to the PTX+VEH group of mice (Figure 5B). Next, 106 miRNAs that showed altered expressions in the PTX+TCDD group were analyzed using Ingenuity Pathway Analysis (IPA) software from QIAGEN. IPA pathway analysis showed several miRNAs that targeted the expression of cytokines, transcription factors, and signaling molecules related to inflammation (Figure 5C). A few examples of the miRNA that targeted inflammatory pathways included: miR-1224-3p and miR-142-3p that targeted the TGF-b pathway, miR-3082-5p that targeted IL-17, and several miRs (miR-671-5p, miR-505-5p, miR-27a-5p, miR-5112, miR-146a-5p) that targeted IL-10 (Figure 5C). Using Affymetrix Expression Console software, heat map of miRNAs of both groups was generated (Figure 5D). In addition, we analyzed miRNA-specific target genes using TargetScan and microRNA.org^1^ analytical tools available online. A list of target genes and their putative 3′UTRs (mRNAs/miRNAs) has been presented in Table 3 with either context++ scores/percentages from TargetScan (Agarwal et al., 2015) or miSVRs from microRNAorg (Peterson et al., 2014). We identified miR-142-3p to target FoxP3, TGFβ2, and TGFβR1, miR-141-3p to target TGFβ2 and TGFβR1, miR-211-3p to target TGFβ1, TGFβ2, TGFβR1, and Smad2 (Table 3). These data suggested that TCDD causes the altered expression of miRNA in T cells that target genes involved in inflammatory pathways.

**TABLE 3:** 3′ UTR alignments and scores of miRNAs and their target genes.

**IL-17**	
5′…AUAAUUUAGCUCCCUACUCUGUU… (Position 585-591)	TargetScan context++ score: −0.26
**| | | | | | |**	
3′ UGUGUCUGUGUGUGUGAGACAG mmu-miR-3082-5p	TargetScan context++ score percentile: 97
**IL-17AR**	
5′…GAGGGUGUAUAUUGUACUCUGUG… (Position 38-44)	TargetScan context++ score: −0.04
**| | | | | | |**	
3′ UGUGUCUGUGUGUGUGAGACAG mmu-miR-3082-5p	TargetScan context++ score percentile: 81
**FoxP3**	
5′ …CAUGAUAGUGCCUGUGUCCUCAA… (Position 1529-1536)	TargetScan context++ score: −0.16
**| | | | | | |**	
3′ GAGGUGGAGGGGUCAGGAGUG mmu-miR-1224-5p	TargetScan context++ score percentile: 91
3′ agguauuucauccuuuGUGAUGu 5’ mmu-miR-142-3p	mirSVR score: −0.0083
**| | | | | | |**	
1737:5′ gccauucccccuuuucCACUACu 3’ FoxP3	PhastCons score: 0.4973
**TGFβ1**	
5′…GUCAGGUGUGUGGCUGUCCUUGA… (Position 1529-1536)	TargetScan context++ score: −0.03
**| | | | | | | | | |**	
3′ CGGGGGGAAACGACAGGAACG mmu-miR-211-3p	TargetScan context++ score percentile: 68
**TGFβR1**	
5′…UGACAUUUUUCCACUUCCUUGAG… (Position 1529-1536)	TargetScan context++ score: −0.02
**| | | | | |**	
3′ CGGGGGGAAACGACAGGAACG mmu-miR-211-3p	TargetScan context++ score percentile: 45
5′ …UUAAAUUUCAUCCUAACACUACA… (Position 1529-1536)	TargetScan context++ score: −0.44
**| | | | | | |**	
3′ AGGUAUUUCAUCCUUUGUGAUGU mmu-miR-142-3p	TargetScan context++ score percentile: 95
5′…UUUAUUUGAUCAAAGCAGUGUUU…(Position 1050-1056)	TargetScan context++ score: −0.47
**| | | | | | |**	
3′ GGUAGAAAUGGUCUGUCACAAU mmu-miR-142-3p	TargetScan context++ score percentile: 98
**TGFβ2**	
5′…GGAGUUUUGAUUCAUCAGUGUUU… (Position 106-112)	TargetScan context++ score: −0.3
**| | | | | | |**	
3′ GGUAGAAAUGGUCUGUCACAAU mmu-miR-141-3p	TargetScan context++ score percentile: 93
5′ …CUAGAUUUUGACUUGCACUACAA… (Position 1222-1228)	TargetScan context++ score: −0.17
**| | | | | |**	
3′ AGGUAUUUCAUCCUUUGUGAUGU mmu-miR-142-3p	TargetScan context++ score percentile: 76
5′…CUUAUCUGAGGAGCUGUCCUUGA… (Position 2098-2105)	TargetScan context++ score: −0.13
**| | | | | | | | | |**	
3′ CGGGGGGAAACGACAGGAACG mmu-miR-211-3p	TargetScan context++ score percentile: 89
**Smad2**	
5′…UGGAUUAACUUGGAAGUCCUUGA… (Position 581-588)	TargetScan context++ score: −0.15
**| | | | | | | | | |**	
3′ CGGGGGGAAACGACAGGAACG mmu-miR-211-3p	TargetScan context++ score percentile: 91

### Validation of miRNAs by Quantitative Real-Time PCR (qRT-PCR)

After analyzing miRNA profiles obtained from miRNA arrays, we selected several miRNAs, those that particularly target cytokines production and Tregs induction, to validate their expression by qRT-PCR. We used the same RNAs from CD4+ T cells that we used for miRNA arrays. We validated the microarray data using qRT-PCR of some select miRs that were up or down-regulated following TCDD treatment. The data demonstrated that there was a significant downregulation of miR-141-3p (Figure 5E, miR142-3p (Figure 5F), and miR-211-3p (Figure 5G) in splenic CD4 T cells from the PTX+TCDD group when compared to the PTX+VEH group.

### qRT-PCR to Determine the Expression miRNA Target Genes

**FIGURE 6:**
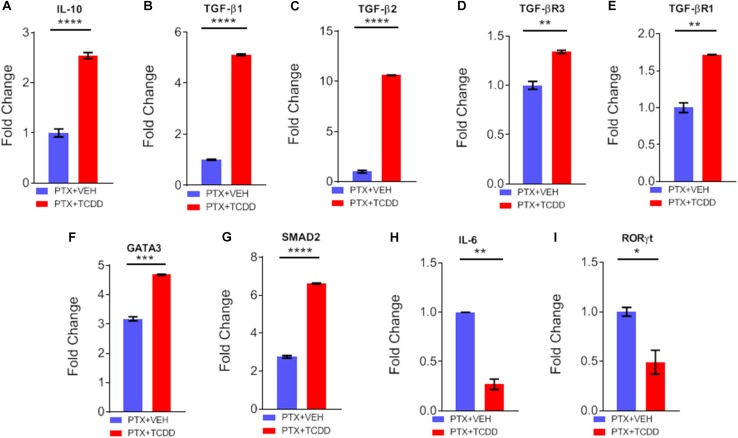
qRT-PCR validation of gene expression. Mice were immunized with PTX and injected with TCDD as described in the legend of Fig 1. Expression of target genes was studied in CD4+ T cells using qRT-PCR in CD4+ T cells: **(A)** IL-10, **(B)** TGF-β1, **(C)** TGFβ2, **(D)** TGFβR3, **(E)** TGFβR1, **(F)** GATA3, **(G)** SMAD2, **(H)** IL-6, and **(I)** RORγt are shown, using GAPDH as a control. Data present mean ± SEM of three experiments and statistical significance between the two groups was tested by a Student’s *t*-test and *p* values were indicated as follows: ^∗∗^*p* < 0.05, ^∗∗∗^*p* < 0.01, and ^****^p < 0.000.

Next, we validated the gene expression of some of the targets of miRs disrupted by TCDD. We noted a significant upregulation of IL-10 (Figure 6A), TGF-β1 (Figure 6B), TGF-β2 (Figure 6C), TGF-βR3 (Figure 6D), TGF-βR1 (Figure 6E), GATA3 (Figure 6F), and SMAD2 (Figure 6G) in cells from mice exposed to PTX+TCDD, when compared to the PTX+VEH group. Additionally, there was significant downregulation of IL-6 (Figure 6H), and RORγT (Figure 6I) in the PTX+TCDD group when compared to the PTX+VEH group.

### Transfection Studies to Demonstrate the Specificity of miR and Their Inflammatory Target Genes

**FIGURE 7:**
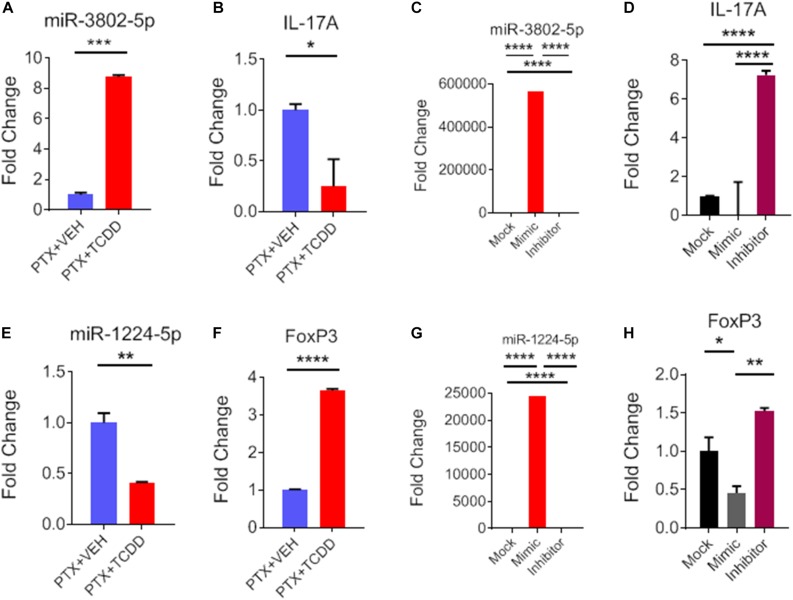
Role of miR-3082-5p and miR-1224-5p in regulating expression of IL-17A and FoxP3, respectively. CD4+ T cells purified from splenocytes form PTX+VEH or PTX+TCDD treated mice were analyzed as described in Figure 6. **(A,B,E,F)**: miRNA and target gene expression were analyzed by qRT-PCR. For transfection studies **(C,D,G,H)**, splenic CD4+ T cells were purified from naive B6 mice and activated by Staphylococcal enterotoxin B (SEB) then transfected with mock, mimic or inhibitor for miR-3082-5p and same for miR-1224-5p. The expression of miRNA and target genes were analyzed using qRT-PCR, and data were expressed as fold change compared to mock controls. Data are presented as the mean ± SEM. The experiments were performed three times with consistent results. Statistical significance between the two groups **(A–C, E–G)** was compared by Student’s *t*-test and panels **D,H** using ANOVA, with *p* values indicated as follows: ^∗^*p* < 0.05, ^∗∗^*p* < 0.01, ^∗∗∗^*p* < 0.001, and ^****^p < 0.0001.

During IPA analysis and using a target scan, we observed that miR-1224-5p and miR-3082-5p had strong binding affinity with 3′UTRs of FoxP3 and IL-17, respectively (Table 3). To confirm that miR-1224 and miR-3082 specifically targeted FoxP3 and IL-17 gene expression, respectively, we first performed qRT-PCR analysis of the expression of both miRs and potential targets from the same cell population namely, splenic CD4+ cells from PTX+VEH or PTX+TCDD. We noted a significant upregulation of miR-3082-5p (Figure 7A) and downregulation of miR-1224 (Figure 7E) in the PTX+TCDD group when compared to the controls. Additionally, in the same cells, there was a significant downregulation of IL-17 expression (Figure 7B) and an increase in FoxP3 expression (Figure 7F) in the PTX+TCDD groups when compared to the controls. These data demonstrated that in the same cells, the miRNA expression induced by TCDD correlated with the expression of respective target genes involved in inflammation.

To further confirm the role of miR-1224-5p and miR-3082-5p in the regulation of FoxP3 and IL-17, respectively, CD4+ T cells were transfected with either miR-1224-5p or miR-3082-5p mimics or their respective inhibitors. Expression of miR-1224-5p and miR-3082-5p was determined by qRT-PCR. There was significant increase in the expression of miR-3802-5p and miR-1224-5p in cells transfected with miR-3082-5p mimic or miR-1224-5p mimic when compared to mock controls (Figures 7C,G). Inhibitors of miR-3082-5p and miR1224-5p completely blocked the expression of these miRs, respectively (Figures 7C,G). In these cells, transfection with miR-3082-5p mimic led to a significant downregulation of IL-17 while the inhibitor caused an upregulation of IL-17 (Figure 7D). Furthermore, transfection of these cells with miR-1224-5p mimic led to the downregulation of FoxP3 while the inhibitor caused an increased expression of FoxP3 (Figure 7H). The data obtained from the transfection experiment demonstrated a clear role of miR-1224-5p and miR-3082-5p in the expression of FoxP3 and IL-17 mediated by exposure to TCDD.

## Discussion

*Bordetella pertussis* is a human respiratory pathogen that causes whooping cough, and its virulence factor, PTX, promotes colonization leading to systemic disease (Connelly et al., 2012). Experimentally, PTX is well-characterized as an adjuvant linked to the development of tissue-specific experimental autoimmune diseases such as experimental autoimmune encephalomyelitis (EAE) and experimental autoimmune uveitis (EAU) (Su et al., 2001; Mohajeri et al., 2015). PTX in these models, is known to enhance vascular permeability, damage to the blood-brain barrier and induces Th1 responses (Hou et al., 2003; Schellenberg et al., 2012). More recent studies suggest that PTX can induce a robust Th17 response while inhibiting Tregs (Chen et al., 2007). While the precise mechanisms through which PTX serves as a virulence factor in promoting *B. pertussis* infection is unclear, it is believed that the ability of PTX, at peak of *B. pertussis* growth in the airways, triggers increased inflammation leading to pathology in the airways (Carbonetti, 2015). Thus, agents that suppress such inflammation induced by PTX may help attenuate the pathogenesis. In the current study, therefore, we tested the effect of TCDD, a potent AhR ligand on PTX-mediated inflammation. Because the effect of AhR activation on a potent toxin such as PTX, known for its ability to promote a Th1 and Th17 response while also suppressing Tregs, has not been studied previously, we were interested to see if TCDD could reverse these effects and if so, to identify the mechanisms.

TCDD is an environmental pollutant with a very high affinity for AhR, a ligand-dependent transcription factor and member of the basic helix-loop-helix-PER-ARNT-SIM gene family (Brunnberg et al., 2003; Ruegg et al., 2008). It is well-characterized for its ability to modulate the immune system (Singh et al., 2011, 2016; Li et al., 2016). More recently, activation of AhR by TCDD has been shown to regulate differential expression of FoxP3 and IL-17, causing generation of more Tregs but suppression of Th17 cells (Quintana et al., 2010; Singh et al., 2011). Studies from our lab have shown that TCDD attenuates colitis in mice by suppressing the expression of IL-17 while promoting that of FoxP3 through epigenetic modifications (Singh et al., 2011). PTX, on the other hand, has been shown to promote the generation of Th17 cells (Chen et al., 2007). Because of the opposite actions of PTX and TCDD in the induction of Th17 and Tregs, we investigated whether TCDD can suppress PTX-induced inflammation in mice and examined the molecular mechanisms involved.

In this study, we noted that injection of PTX triggered significant systemic inflammation in mice as evidenced by the detection of several inflammatory cytokines in the serum including IL-17, IL-6, and IFNγ. These cytokine expressing cells were also detected in CD4+ T cells in the spleen. Interestingly, the serum levels of IL-17 or proportion of Th17 cells in the spleens of PTX-treated mice was higher than that of IFN-γ or Th1 cells in the spleen, thereby demonstrating that PTX is a more potent inducer of Th17 cells than Th1 cells. These data were consistent with the observation that PTX generates a cytokine storm associated with inflammation in mice by inducing IL-6 which promotes IL-17 production in CD4+ T cells (Chen et al., 2007). Furthermore, our findings are in agreement with previous reports showing that PTX administration can induce pathogenic Th17 cells in mice with immune-mediated ocular inflammation more than other TLR ligands, such as LPS, and Polyinosinic:polycytidylic acid (Fujimoto et al., 2006). Induction of Th17 cells has also been shown in studies using pertussis vaccination in mice (Bird et al., 1998). Previous studies, using mice infected with PTX–producing *B. pertussis* strain or an isogenic PTX-deficient strain (ΔPT), had shown that PTX activity was associated with upregulated expression of proinflammatory cytokines and chemokines (Andreasen et al., 2009; Connelly et al., 2012). Studies on immunomodulation by *B. pertussis* have also shown that the host immune response may be tilted toward development Th17 cells that produce IL-17A.

In the current study, we noted that TCDD caused a significant decrease in inflammatory (IL-17, IL-6, and IFN-γ) cytokines but an increase in anti-inflammatory (IL-10, TGF-b) cytokines in PTX-injected mice. IL-10 is an anti-inflammatory cytokine that is known to inhibit the activity of Th1 cells (Couper et al., 2008). TGF-b inhibits Th1 differentiation by blocking the expression of T-bet, the master regulator of Th1 cell differentiation (Gorelik et al., 2002). We also noted that TCDD was able to induce a switch from Th17 to Tregs in PTX-immunized mice. This may result from decreased IL-6 seen in PTX+TCDD treated mice because IL-6-mediated STAT3 signaling is essential for Th17 differentiation and plays a central role in the pathogenesis of certain autoimmune diseases such as rheumatoid arthritis (Park et al., 2014). The second mechanism through which TCDD may suppress Th17 cells may result from its ability to increase the expression of FoxP3 and to induce more Tregs, as seen in the current study using PTX and as seen in other autoimmune disease models (Kerkvliet et al., 2009; Benson and Shepherd, 2011). For example, TCDD-mediated Treg induction in pancreatic lymph was shown to suppress diabetes in NOD mice (Kerkvliet et al., 2009). In addition, induction of Tregs by TCDD lessened EAE-associated disease burden (Quintana et al., 2008). Because EAE mouse models use PTX along with myelin oligodendrocyte glycoprotein (MOG) to enhance clinical signs of EAE, our studies raise an interesting question on whether TCDD-mediated suppression of EAE results from suppression of PTX-mediated effects or by direct action on MOG-specific T cells. Similarly, previous reports from our lab showed that Treg-specific (FoxP3) and Th17-specific (IL-10) genes were epigenetically-regulated in a mouse model of colitis, leading to decreased disease severity (Singh et al., 2011). In this report, we noted that there was decreased methylation of CpG islands of Foxp3 and increased methylation of IL-17 promoters, which led to the increased induction of Tregs while suppressing Th17 generation (Singh et al., 2011). In the current study, we noted that TCDD induced MDSC in PTX-treated mice. Because Tregs have also been shown to be induced by MDSCs (Nakamura and Ushigome, 2018), this may constitute another additional mechanism through which Tregs were induced in PTX+TCDD administered mice. As there were more Tregs in PTX+TCDD-treated mice, it is possible that the increased IL-10 seen in these mice may have resulted from such Tregs producing IL-10 (Chaudhry et al., 2011).

In recent years, miRs have been shown to play an important role in the regulation gene expression and may potentially control the majority of molecular and cellular pathways (Grimm et al., 2006; Tsuchiya et al., 2006; Gaur et al., 2007). Furthermore, specific miRs may play important roles in T cell and MDSCs development and functions by regulating target genes involved in their proliferation and differentiation (Hegde et al., 2013). Previous studies from our lab have shown that AhR activation through dietary ligands such as indole-3-carbinol (I3C) or 3,3′-diindolylmethane (DIM) leads to decreased the expression of several miRs (miR-31, miR-219, and miR-490) that target Foxp3, while increasing the expression of miR-495 and miR-1192 that target IL-17 (Singh et al., 2016). This leads to an increased induction of Tregs and decreased differentiation of Th17 cells consequently suppressing a delayed-type hypersensitivity (DTH) response to methylated BSA (Singh et al., 2016). In another study, we noted that miRNA-466a-3p targeted TGF-β2 and regulated Treg differentiation (Becker et al., 2018). Additional studies from other groups have also shown that AhR activation, even with other AhR ligands, can regulate miR expression as well. For example, the activation of AhR by berberine stimulated the increase of miR-21-3p in a breast cancer cell line (Lo et al., 2017). AhR knockout mouse studies in chronic cigarette smoke exposure models revealed this receptor was important in the regulation of several pulmonary-associated miRs (Rogers et al., 2017). TCDD and a selective modulator of AhR (6-methyl-1,3,8-trichlorodibenzofuran, MCDF) were also shown to inhibit lung metastasis through the induction of miR-335 (Zhang et al., 2012). In the current study, we observed that the PTX+TCDD group showed significant alterations in the expression of the numbers of miRNAs and pathway analysis showed that some of these altered miRNAs targeted inflammatory cytokines or signaling pathways. Interestingly, we were able to narrow down to some specific miRs that seemed to target FoxP3 and IL-17 gene expression through target scanning. Specifically, we found that miR-1224-5p and miR-3082-5p had strong binding affinity with 3′UTRs of FoxP3 and IL-17, respectively. We confirmed the role of miR-1224-5p and miR-3082-5p in the regulation of FoxP3 and IL-17, respectively, by performing transfection studies which conclusively demonstrated that miR-1224-5p and miR-3082-5p were involved, at least in part, in the upregulation of FoxP3 and the down regulation of IL-17 following exposure to TCDD. The role of miR-1224-5p in the regulation of immune response has not been previously studied. miR-1224-5p has been shown to act as a tumor suppressor by targeting CREB1 in malignant gliomas (Qian et al., 2015). Similarly, the role of miR-3082-5p has not been reported in any disease model. Thus, our studies are unique in identifying for the first time that miR-1224-5p and miR-3082-5p play a role in the regulation of inflammation. While we focused on these two miRs, we also noted that several other miRs may also be involved in attenuating inflammatory signaling pathways.

In summary, our studies suggest that during PTX-induced inflammation, activation of AhR by TCDD may promote anti-inflammatory activity through regulation of miRNA that target Foxp3 and IL-17 genes. Because PTX plays a critical role in the pathogenesis of *B. pertussis* infection, by inducing cytokine storm and exaggerated inflammatory response, our studies suggest that AhR ligands may serve as a therapeutic modality to treat such inflammatory response.

## Data Availability Statement

Raw data from the microRNA array can be accessed at the Gene Expression Omnibus (GEO) repository with accession number GSE137980.

## Ethics Statement

The animal study was reviewed and approved by Institutional Animal Care and Use Committee (IACUC) of the University of South Carolina School of Medicine.

## Author Contributions

ZA-G, MN, and PN: conceptualization, funding acquisition and resources. ZA-G, PM-B, MN, and PN: methodology. ZA-G: validation and visualization. ZA-G and PB: formal analysis and writing- original draft. ZA-G and PM-B: investigation. ZA-G, NS, PB, MN, and PN: writing, review and editing. MN and PN: supervision.

## Conflict of Interest

The authors declare that the research was conducted in the absence of any commercial or financial relationships that could be construed as a potential conflict of interest.

## Footnotes

1 www.microRNA.org
